# Five-year overall survival of early- and late-onset colorectal cancer in Medellín, Colombia: a comparative study

**DOI:** 10.1007/s00432-024-06007-7

**Published:** 2024-11-09

**Authors:** Álvaro Esteban Ruiz-Grajales, Juan Camilo Correa-Cote, Miguel Ángel Sánchez-Zapata, Manuela María Orozco-Puerta, Juan Felipe Baena-García, Esteban Castrillón-Martínez

**Affiliations:** 1 Semillero de Investigación en Salud (SEIS), Facultad de Medicina, Universidad de Antioquia UdeA, St. 51D # 62-29, Medellín, 050010470 Colombia; 2 Clínica Medellín S.A.S, Medellín, Colombia; 3 Department of Surgery, Facultad de Medicina, Universidad de Antioquia UdeA, Medellín, Colombia; 4 Semillero de Investigación en Medicina Interna (SIMI), Facultad de Medicina, Universidad de Antioquia UdeA, Medellín, Colombia; 5 Hospital Alma Máter de Antioquia, Medellín, Colombia

**Keywords:** Colorectal neoplasms, Survival analysis, Age of onset, Epidemiology, Colombia

## Abstract

**Purpose:**

Early-onset colorectal cancer (CRC) (EOCRC, < 50 years) has distinct clinicopathological features from late-onset CRC (LOCRC, ≥ 50 years). However, evidence on survival outcomes is contradictory. We aimed to analyse the differences in 5-year overall survival (OS) between EOCRC and LOCRC.

**Methods:**

A retrospective cohort study was conducted during 2018–2022. Individuals aged ≥ 18 years diagnosed with CRC at two hospitals in Medellín, Colombia were included. Clinicopathological and survival data were retrieved from the medical records and a public government database. Patients were categorized into EOCRC and LOCRC groups. Five-year OS rates were calculated using the Kaplan-Meier method and prognostic factors for OS were identified through Cox regression models.

**Results:**

Among 1022 patients, 52.5% were female, and 13.5% (*n =* 138) had EOCRC. Patients with EOCRC showed higher 5-year OS rates than LOCRC patients (54% vs. 32%). Univariable analyses indicated a 37% lower risk of death for EOCRC compared to LOCRC (HR: 0.633, 95%CI: 0.476–0.840, *p* = 0.002). After multivariable analyses, advanced staging and higher tumour grading were prognostic factors for worse OS (HR: 2.127, 95% CI:1.405–3.220, *p* = 0.0001; and HR: 12.896, 95%CI: 6.310-26.355, *p* = 0.000; respectively), and being in the EOCRC group remained as a prognostic factor for higher OS (HR: 0.482, 95% CI: 0.336–0.690, *p* = 0.000).

**Conclusion:**

EOCRC is associated with significantly better 5-year OS rates and prognosis compared to LOCRC. Advanced stage and higher tumour grading are predictors of lower OS among all CRC patients. These findings highlight the importance of age-related risk stratification and personalized therapeutic approaches in CRC.

## Introduction

Colorectal cancer (CRC) is one of the top malignant neoplasms for both sexes worldwide (Siegel et al. 2023). Over the last few decades, early-onset CRC (EOCRC, CRC in persons < 50 years of age) has been increasing in numerous countries compared to late-onset CRC (LOCRC, ≥ 50 years of age) (Sinicrope 2022). Although the underlying cause has not been fully elucidated, evidence indicates that EOCRC is clinical and pathologically different than LOCRC (Muller et al. 2021). However, data regarding differences in survival outcomes between EOCRC and LOCRC are contradictory.

Several global studies, when examined without considering factors such as cut-off ages, staging, tumour site and treatment, have concluded that EOCRC has better survival outcomes compared to LOCRC (Rodriguez et al. 2018; Aguiar-Junior et al. 2020; Chen et al. 2020; Saraste et al. 2020; Zaborowski et al. 2020; Alvarez et al. 2021; Griffiths et al. 2021; Alsiary et al. 2023; Jeri-Yabar et al. 2024; Ren et al. 2024). However, other investigations have found no differences in survival outcomes as well (Wan Ibrahim et al. 2020; Wong et al. 2021; Gao et al. 2022; Lipsyc-Sharf et al. 2022; Park et al. 2022, 2023; Swartjes et al. 2022; Al Zaabi et al. 2023; Rashad et al. 2024). Furthermore, some studies have reported that EOCRC is, in fact, associated with lower survival rates than LOCRC (Fontana et al. 2021; Tang et al. 2021; Foppa et al. 2022; McClelland et al. 2022).

Proposed explanations for younger patients having a better prognosis than their older counterparts include some characteristics such as having fewer or no comorbidities, a more resilient profile to aggressive therapies due to better physiological reserves, the likelihood of completing treatment regimens, and age itself (REACCT Collaborative et al. 2021; Eng et al. 2022). Conversely, other authors argue that the prognosis for EOCRC is poorer, primarily because it tends to present at an advanced stage with a more aggressive tumour profile compared to LOCRC (Muller et al. 2021).

CRC survival has been scarcely studied in Colombia (Jurado-Fajardo et al. 2011; Cortés et al. 2014; Pardo and de Vries 2017; Campo-Sánchez et al. 2019; Lema et al. 2021; Buitrago et al. 2022; Guzmán-Gallego et al. 2023; Preciado-Franco et al. 2023). Likewise, the number of studies focusing on the survival differences between EOCRC and LOCRC is low (Jurado-Fajardo et al. 2011; Cortés et al. 2014; Guzmán-Gallego et al. 2023). Although these investigations indicate that Colombian adults with EOCRC experience better survival outcomes than adults with LOCRC, evidence on this matter is limited, thus, further research is needed. We aimed to analyse the differences in five-year overall survival (OS) between EOCRC and LOCRC in a large cohort of patients from Medellín, Colombia.

## Methods

### Study design and setting

A retrospective cohort study was carried out in Medellín, Colombia from January 1st, 2018, to December 31st, 2022. Two tertiary hospitals participated. This study used an extension of a sample of patients with CRC previously analysed on their clinical and pathological differences by age of onset (Ruiz-Grajales et al. 2024). This work followed the recommendations of the *STrengthening the Reporting of OBservational studies in Epidemiology* (STROBE) initiative (Von Elm et al. 2008).

### Patient population

Patients aged 18 years or older diagnosed with invasive or non-invasive colon or rectal cancer by histopathology were included. The diagnosis was considered according to the International Classification of Diseases, 10^th^ Ed. (ICD-10) (World Health Organization 2016). Noteworthy, we included the ICD-10 codes C19 (malignant neoplasm of rectosigmoid junction), C18.8 (malignant neoplasm: overlapping lesion of colon) and C18.9 (malignant neoplasm: colon, unspecified). All patients with incomplete electronic medical records, loss of follow-up, metastasis to the colon and/or rectum from another primary tumour, recurrent CRC, history of another primary malignant tumour, and history of a synchronous malignant tumour (defined as a second primary malignant tumour diagnosed within 6 months after the primary colorectal tumour) were excluded.

### Study variables

The electronic medical records were reviewed. The following characteristics were retrieved: age (at the time of diagnosis); sex; comorbidities; inflammatory bowel disease, toxicological and CRC family history; time to diagnosis; clinical signs and/or symptoms; tumour location, histology and grading; pathological TNM stage according to the classification of the American Joint Committee on Cancer (AJCC), 7^th^ Ed (Edge et al. 2010); metastases location; and intent and type of first treatment.

The primary outcome was five-year OS. Patients were retrospectively followed from the month in which they were diagnosed to the month of death (from any cause) or the month of vital status review. Vital status was verified using the public database *Unified Database of Affiliates* (BDUA, for its acronym in Spanish) of the *General System of Social Security in Health* - Ministry of Health and Social Protection of Colombia (Administradora de los Recursos del Sistema General de Seguridad Social en Salud 2024).

To reduce potential bias, two authors (not involved in the data collection) independently verified the information retrieved from random selected patients (*n* = 50). All inconsistencies, if any, were discussed and corrected. Also, the final database was revised for errors and/or missing values.

### Statistical analysis

Missing values were not included in the analyses. Continuous variables were presented in mean and standard deviation or median and interquartile range (IQR), according to data distribution. Frequencies and percentages for categorical variables were calculated.

Two analysis groups were established: EOCRC and LOCRC. The cut-off age for patients in the EOCRC group was 49 years or less, and 50 years or older for the LOCRC group. One-, three-, and five-year OS rates were estimated. Differences in OS between EOCRC and LOCRC were analysed using the Kaplan-Meier method and the Log-Rank test was calculated. We further stratified by tumour location, histology, grading, and staging. Univariable and multivariable Cox proportional hazards regression models were carried out to identify prognostic factors associated with OS. Crude and adjusted hazard ratios with their corresponding 95% confidence intervals were reported.

All *P* values were 2-sided. The significance level was established at less than 0.05. All analyses were conducted using the Statistical Package for the Social Sciences (SPSS), version 25.0.0.0 (IBM) (IBM Corp 2017).

## Results

### Patients characteristics

**Fig. 1:**
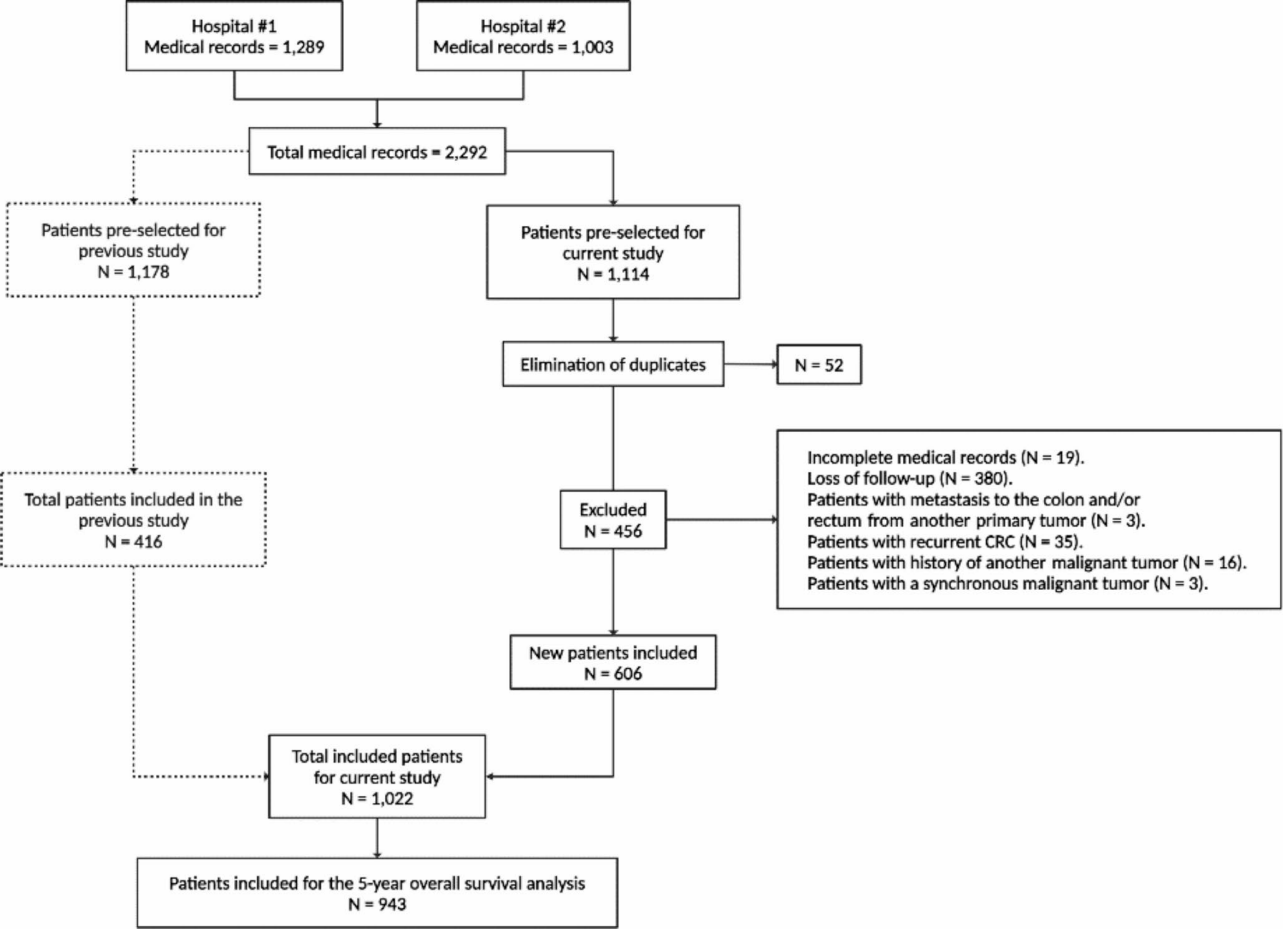
Patient selection flowchart. CRC, colorectal cancer

A cumulative sample of 1022 patients was obtained (Fig. 1). Females comprised 52.5% of the total sample (*n* = 537). The median age for all participants was 66 years (IQR: 56–75). Among them, 13.5% (*n* = 138) belonged to the EOCRC group, with a median age of 40 years (IQR: 35–46). Patients with LOCRC had a median age of 69 years (IQR: 61–76). The characteristics of CRC patients are shown in Table 1.

We observed differences between groups regarding comorbidities, and toxicological and CRC family history. The LOCRC group exhibited a higher prevalence of cardiometabolic diseases compared to the EOCRC group. For instance, arterial hypertension was documented in 50.0% of older patients compared to 6.5% in younger individuals. Additionally, smoking history was more prevalent in LOCRC compared to EOCRC (31.7% vs. 8.7%). Conversely, CRC family history was frequent in EOCRC than in LOCRC (8.7% vs. 3.4%). Furthermore, younger patients had a higher prevalence of time to diagnosis ≥ 6 months (45.1% vs. 34.3%), as well as left colon (34.8% vs. 30.7%) and rectum tumours (37.7% vs. 31.8%). Also, histological subtypes other than adenocarcinoma not otherwise specified (NOS), poorly differentiated tumour grading (10.2% vs. 5.8%), and pN2 staging (40.5% vs. 25.4%) were more prevalent among younger patients.

**Table 1:** Characteristics of adults with colorectal cancer

Characteristic	LOCRC (*n* = 884)	EOCRC (*n* = 138)	Total (*n* = 1022)
Age, median (IQR)	69 (61–76)	40 (35–46)	66 (56–75)
Sex, *n* (%)			
Female	463 (52.4)	74 (53.6)	537 (52.5)
Male	421 (47.6)	64 (46.4)	485 (47.5)
Comorbidities, *n* (%)			
Arterial hypertension	442 (50.0)	9 (6.5)	451 (44.1)
Diabetes mellitus	168 (19.0)	8 (5.8)	176 (17.2)
Dyslipidaemia^a^	138 (15.6)	4 (2.9)	142 (13.9)
Colorectal polyps^b^	22 (2.5)	2 (1.4)	24 (2.3)
Inflammatory bowel disease history, *n* (%)			
Ulcerative colitis	9 (1.0)	0 (0.0)	9 (0.9)
Crohn’s disease	1 (0.1)	0 (0.0)	1 (0.1)
Toxicological history^c^, *n* (%)			
Smoking^d^	280 (31.7)	12 (8.7)	292 (28.6)
Alcohol consumption^e^	121 (13.7)	11 (8.0)	132 (12.9)
CRC family history^f^, *n* (%)	30 (3.4)	12 (8.7)	42 (4.1)
Diagnosis, *n* (%)			
By clinical signs and/or symptoms	735 (97.1)	109 (96.5)	844 (97.0)
Surveillance^g^, incidental or screening	22 (2.9)	4 (3.5)	26 (3.0)
Unknown, *n*	127	25	152
Time to diagnosis^h^, *n* (%)			
< 6 months	405 (65.7)	50 (54.9)	455 (64.4)
≥ 6 months	211 (34.3)	41 (45.1)	252 (35.6)
Unknown, *n*	268	47	315
Clinical signs and symptoms, *n* (%)			
Lower gastrointestinal tract bleeding	376 (51.2)	64 (58.2)	440 (52.1)
Abdominal pain	454 (61.8)	74 (67.3)	528 (62.5)
Weight loss	348 (47.3)	54 (49.1)	402 (47.6)
Bowel habits changes^i^	378 (51.4)	64 (58.2)	442 (52.3)
Bowel obstruction	185 (25.2)	32 (29.1)	217 (25.7)
Anorectal pain^j^	94 (12.8)	21 (19.1)	115 (13.6)
Unknown, *n*	149	28	177
Tumour location, *n* (%)			
Right colon^k^	323 (36.5)	36 (26.1)	359 (35.1)
Left colon^l^	271 (30.7)	48 (34.8)	319 (31.2)
Rectum	281 (31.8)	52 (37.7)	333 (32.6)
Not specified^m^	9 (1.0)	2 (1.4)	11 (1.1)
Tumour histology, *n* (%)			
Adenocarcinoma NOS	802 (90.7)	113 (81.9)	915 (89.5)
Mucinous adenocarcinoma	51 (5.8)	14 (10.1)	65 (6.4)
Signet ring cell carcinoma	9 (1.0)	4 (2.9)	13 (1.3)
Squamous cell carcinoma	6 (0.7)	3 (2.2)	9 (0.9)
Neuroendocrine carcinoma	7 (0.8)	2 (1.4)	9 (0.9)
Other histological subtypes^n^	9 (1.0)	2 (1.4)	11 (1.1)
Tumour grading, *n* (%)			
Well differentiated	465 (59.7)	57 (52.8)	522 (58.9)
Moderately differentiated	269 (34.5)	40 (37.0)	309 (34.8)
Poorly differentiated	45 (5.8)	11 (10.2)	56 (6.3)
Unknown, *n*	105	30	135
Staging, *n* (%)			
I	69 (9.6)	7 (5.6)	76 (9.0)
II	165 (22.9)	29 (23.2)	194 (23.0)
III	249 (34.6)	46 (36.8)	295 (35.0)
IV	228 (31.7)	42 (33.6)	270 (32.0)
Unknown, *n*	173	14	187
Tumour size and extend, *n* (%)			
T in situ	11 (1.5)	1 (0.9)	12 (1.4)
pT1	17 (2.4)	4 (3.5)	21 (2.5)
pT2	92 (12.8)	10 (8.7)	102 (12.2)
pT3	382 (53.1)	57 (49.6)	439 (52.6)
pT4	218 (30.3)	43 (37.4)	261 (31.3)
Unknown, *n*	164	23	187
Regional lymph nodes involvement, *n* (%)			
pN0	293 (42.0)	43 (38.7)	336 (41.6)
pN1	227 (32.6)	23 (20.7)	250 (30.9)
pN2	177 (25.4)	45 (40.5)	222 (27.5)
Unknown, *n*	187	27	214
Metastasis, *n* (%)			
M0	497 (68.6)	83 (66.4)	580 (68.2)
M1	228 (31.4)	42 (33.6)	270 (31.8)
Unknown, *n*	159	13	172
Metastases location, *n* (%)			
Liver	169 (74.1)	22 (52.4)	191 (70.7)
Lungs	84 (36.8)	11 (26.2)	95 (35.2)
Peritoneum	45 (19.7)	11 (26.2)	56 (20.7)
Non-regional lymph nodes	13 (5.7)	3 (7.1)	16 (5.9)
Bone	10 (4.4)	0 (0.0)	10 (3.7)
Intrabdominal organs^o^	14 (6.1)	9 (21.4)	23 (8.5)
Intent of first treatment, *n* (%)			
Curative	611 (71.6)	91 (66.9)	702 (71.0)
Palliative	228 (26.7)	45 (33.1)	273 (27.6)
No treatment	14 (1.6)	0 (0.0)	14 (1.4)
Unknown, *n*	31	2	33
Type of first treatment, *n* (%)			
Surgery^p^	685 (82.1)	96 (70.1)	781 (80.4)
Chemotherapy, radiotherapy, or both	129 (15.5)	40 (29.2)	169 (17.4)
Palliative care	20 (2.4)	1 (0.7)	21 (2.2)
Unknown, *n*	50	1	51

CRC, colorectal cancer; EOCRC, early-onset CRC; LOCRC, late-onset CRC; NOS, not otherwise specified

^a^Includes hypercholesterolemia and/or hypertriglyceridemia

^b^All type of polyps, including advanced adenoma

^c^Current or previous, time exposure was not considered

^d^Only exposure to tobacco was considered

^e^All types of alcohol-containing beverages were considered

^f^First- or second-degree relatives

^g^Clinical follow-up due to history of inflammatory bowel disease or colorectal polyps

^h^Time between onset of clinical signs and/or symptoms and histopathological diagnosis

^i^Constipation, diarrhoea, or both

^j^Includes rectal tenesmus, rectal straining, sensation of mass in the rectum, anal burning sensation, and dyschezia

^k^Cecum, ascending colon, hepatic flexure, and transverse colon

^l^Splenic flexure, descending colon, sigmoid colon and rectosigmoid junction

^m^C18.9 diagnosis (malignant neoplasm: colon, unspecified)

^n^Colorectal sarcoma, colorectal lymphoma, and undifferentiated carcinoma

^o^Includes duodenum, pancreas, bladder, abdominal wall, biliary tree, stomach, omentum, spleen, kidney, adrenal glands, ureter, and retroperitoneum

^p^Includes tumour resection as well as derivative surgery (colostomy), endoscopic microsurgery, and polypectomy

### Overall survival

**Fig. 2:**
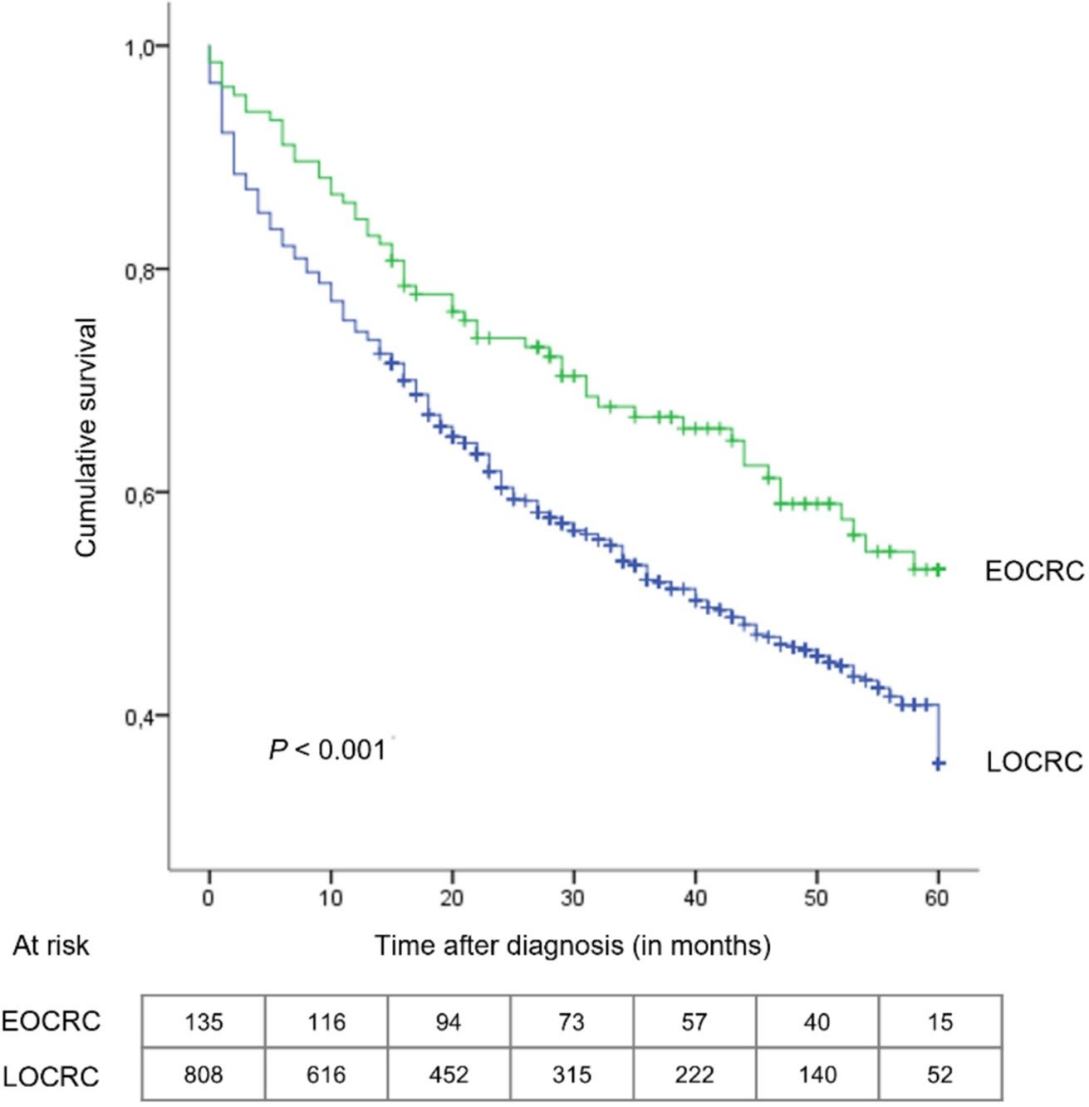
Kaplan-Meier overall survival curves of patients with colorectal cancer stratified by age of onset. EOCRC, early-onset colorectal cancer; LOCRC, late-onset colorectal cancer

Data for the survival analyses were available for 92.2% (*n* = 943) of the sample. Median follow-up was 38 months for the EOCRC group and 25 months for the LOCRC group. One-, three- and five-year OS rates for both groups were estimated (Table 2). The EOCRC group exhibited higher OS rates in all time points. Median 5-year OS was only reached in the LOCRC group: 41 months (95%CI: 34.568–47.432). The Log-Rank test indicated statistically significant OS differences between EOCRC and LOCRC (Fig. 2).

**Table 2:** Overall survival rates of adults with colorectal cancer

Overall survival	LOCRC (*n* = 808)	EOCRC (*n* = 135)	Total (*n* = 943)
One-year, %	62	74	63
Three-year, %	46	59	48
Five-year, %	32	54	35

EOCRC, early-onset colorectal cancer; LOCRC, late-onset colorectal

**Fig. 3:**
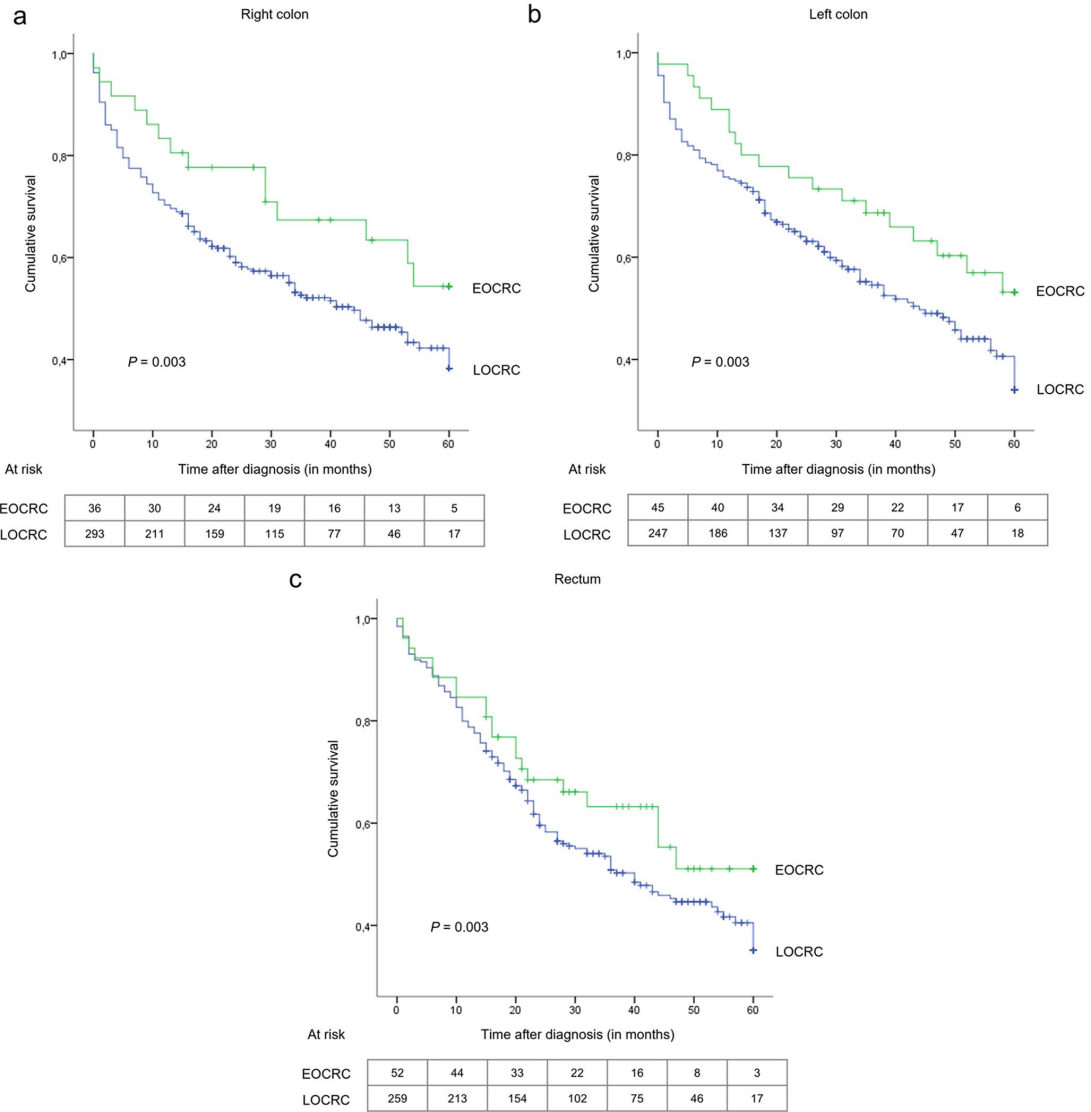
Kaplan-Meier overall survival curves of patients with early- and late-onset colorectal cancer stratified by tumour location: (**a**) right colon, (**b**) left colon, (**c**) rectum. EOCRC, early-onset colorectal cancer; LOCRC, late-onset colorectal cancer

**Fig. 4:**
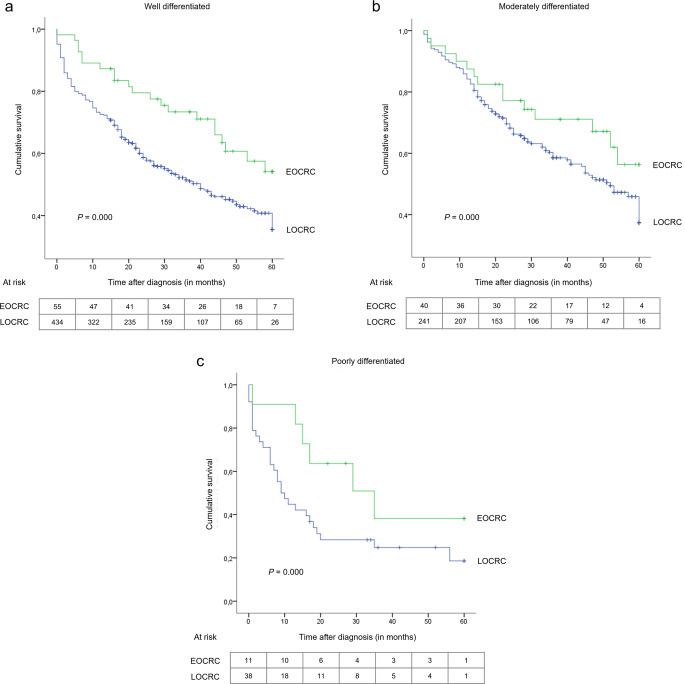
Kaplan-Meier overall survival curves of patients with early- and late-onset colorectal cancer stratified by tumour grading: (**a**) well differentiated, (**b**) moderately differentiated, (**c**) poorly differentiated. EOCRC, early-onset colorectal cancer; LOCRC, late-onset colorectal cancer

When stratified by tumour location (Fig. 3) and grading (Fig. 4), differences in the distribution of OS rates between groups remained statistically significant. This was not the case for tumour histology and staging.

### Prognostic factors associated with overall survival

Univariable Cox regression analyses revealed that poorly differentiated tumour grading (HR: 1.812; 95% CI: 1.271–2.582; *p* = 0.001) and stage IV (HR: 13.077; 95% CI: 6.440-26.557; *p* = 0.000) were associated with lower 5-year OS. Conversely, earlier age at diagnosis (HR: 0.633; 95% CI: 0.476–0.840; *p* = 0.002) and moderately differentiated tumour grading (HR: 0.796; 95% CI: 0.643–0.986; *p* = 0.037) were associated with better 5-year OS (Table 3). After multivariable Cox regression analyses, poorly differentiated tumour grading (aHR: 2.127; 95% CI: 1.405–3.220; *p* = 0.001) and stage IV (aHR: 12.896; 95% CI: 6.310-26.355; *p* = 0.0) persisted as significantly associated with inferior 5-year OS. Moreover, being in the EOCRC group remained as the only variable correlated with better 5-year OS after adjusting for tumour features and staging (aHR: 0.482; 95% CI: 0.336–0.690; *p* = 0.0).

**Table 3:** Univariable and multivariable Cox regression analyses of prognostic factors associated with overall survival in adults with colorectal cancer

Characteristic	Univariable analysis			Multivariable analysis		
	HR	95%CI	*P* value	aHR	95%CI	*P* value
Group						
LOCRC	*Ref*.	―	―	*Ref*.	―	―
EOCRC	0.633	0.476–0.840	*0.002	0.482	0.336–0.690	*0.000
Tumour location						
Right colon	*Ref*.	―	―	*Ref.*	―	―
Left colon	0.943	0.754–1.180	0.610	1.034	0.782–1.366	0.817
Rectum	0.837	0.977 − 0.785	0.837	1.104	0.839–1.454	0.479
Tumour histology						
Adenocarcinoma NOS	*Ref*.	―	―	*Ref*.	―	―
Other histological subtypes^a^	1.185	0.896–1.566	0.234	1.084	0.724–1.622	0.695
Tumour grading						
Well differentiated	*Ref*.	―	―	*Ref*.	―	―
Moderately differentiated	0.796	0.643–0.986	*0.037	0.833	0.655–1.060	0.138
Poorly differentiated	1.812	1.271–2.582	*0.001	2.127	1.405–3.220	*0.000
Staging						
I	*Ref*.	―	―	*Ref.*	―	―
II	2.719	1.291–5.724	*0.008	2.704	1.274–5.740	*0.010
III	3.487	1.700-7.155	*0.001	3.598	1.748–7.405	*0.001
IV	13.077	6.440-26.557	*0.000	12.896	6.310-26.355	*0.000

EOCRC, early-onset colorectal cancer; LOCRC, late-onset colorectal cancer; NOS, not otherwise specified

^a^Mucinous adenocarcinoma, signet ring cell carcinoma, squamous cell carcinoma, neuroendocrine carcinoma, colorectal sarcoma, colorectal lymphoma, and undifferentiated carcinoma

*Statistically significant

## Discussion

In Colombia, there is a lack of studies comparing survival differences between EOCRC and LOCRC. To address this gap, we conducted a cohort study of CRC patients in Medellín, Colombia, with the aim of analysing their OS disparities. Our study indicates that EOCRC is associated with a higher 5-year OS, whereas advanced stages and higher tumor grading are linked to lower 5-year OS among all CRC patients. Specifically, the differences in the 5-year OS rates were statistically significant, with 54% for EOCRC and 32% for LOCRC. This suggests that among Colombian adults with CRC, a higher proportion of EOCRC cases survive five years post-diagnosis compared to LOCRC cases.

Supporting this finding, a study from Brazil reported a 5-year OS rate of 70.0% for adults with EOCRC (< 50 years), while those aged 50–74 and ≥ 75 years had lower rates of 66.9% and 43.8%, respectively (Aguiar-Junior et al. 2020). In contrast, a study conducted with Chilean patients showed that younger adults (≤ 50 years) had a lower 5-year OS rate (73.6%) than adults in the 51–69 years group (80%). However, the late-onset group (≥ 70 years) had the lowest survival rate (48.5%) (Alvarez et al. 2021). These regional disparities are particularly interesting given the geographical proximity of the study populations.

After adjusting for tumor site and grading, the differences in 5-year OS rates remained higher for younger individuals in our sample. For example, EOCRC patients with right-sided tumours experienced better OS rates than their counterparts with the same tumor location (Fig. 3A). Our findings suggest that age is associated with a better prognosis, regardless of tumor site. However, it is important to note that tumor site was not an independent prognostic factor for OS after Cox regression analyses in our study. Other investigations have reported conflicting results. For instance, one study found that tumor sidedness is not an independent prognostic factor for OS among staged I-III EOCRC patients (Azar et al. 2021). Conversely, some authors have stated that right-sided tumours are associated with poorer overall and CRC-specific survival due to their histopathological profile, regardless of age (Akimoto et al. 2021; Di Leo et al. 2021; REACCT Collaborative et al. 2021; Dharwadkar et al. 2022).

Our study also demonstrated that advanced staging at diagnosis is associated with poorer oncological outcomes among CRC patients. Within our sample, individuals with stage IV were approximately 12 times more likely to die within the first five years than those diagnosed at stage I. Metastatic disease is strongly associated with higher morbidity and mortality rates, as well as an increased incidence of both local and distant recurrences (Hernandez-Dominguez et al. 2023). Although we did not find significant differences in tumor staging between EOCRC and LOCRC, it has been reported that EOCRC is frequently diagnosed at more advanced stages (REACCT Collaborative et al. 2021). This is thought to be due to a higher prevalence of genetic or underlying familial factors that contribute to faster cancer progression, bypassing the traditional adenoma-carcinoma sequence, which can take up to 15 years to develop (Muller et al. 2021). Interestingly, observational studies have shown that, despite presenting at advanced stages, younger CRC patients exhibit higher survival rates compared to older patients (Di Leo et al. 2021). One plausible explanation for this discrepancy is that EOCRC may behave differently from LOCRC in several ways. For instance, younger patients might have a better response to treatment regimens and greater physiological reserves, which could contribute to their improved survival outcomes (REACCT Collaborative et al. 2021).

Furthermore, our study found that tumor grading is an independent prognostic factor for worse OS among all individuals. This linkage has been reported previously (REACCT Collaborative et al. 2021). However, evidence on age-related differences suggests no clear association. EOCRC often presents with poorly differentiated tumours and non-adenocarcinoma NOS histology (e.g., signet-ring cell, mucinous), which are associated with a more treatment-resistant disease and higher rates of local and distant recurrences (REACCT Collaborative et al. 2021). Despite this, younger patients are more likely to receive aggressive surgical and systemic therapies earlier and with longer duration, despite the increased incidence of toxic side effects (Done and Fang 2021; Dharwadkar et al. 2022).

Our study highlights the role of younger age as a prognostic factor for OS in CRC patients. In our sample, patients with EOCRC were approximately 37% less likely to die from any cause within the first five years compared to those with LOCRC. After adjusting for tumor characteristics and staging, this increased to 52%. Supporting our findings, researchers have reported that patients aged 50–74 and ≥ 75 years had a significantly higher risk of death (HR: 1.24; 95% CI: 1.02–1.51 and HR: 3.02; 95% CI: 2.42–3.78, respectively) compared to younger adults (< 50 years) (Aguiar-Junior et al. 2020). Furthermore, in Colombia, three studies have concluded that younger CRC patients experience better survival outcomes than older CRC patients. A population-based study conducted in Manizales, Colombia found that patients > 75 years old had lower CRC-specific survival than adults < 50 years of age (HR: 1.97, 95%CI: 1.44–2.69) (Guzmán-Gallego et al. 2023). Similar results were found in CRC patients from Cali, Colombia, as older persons (≥ 70 years) had around a 145% increment in their risk of dying from CRC than younger individuals (< 50 years) (HR: 2.45, standard error: 0.23, *p =* 0.00) (Cortés et al. 2014). Moreover, another study reported that adults with CRC in Pasto, Colombia aged 35–54 years were 60% less likely to die from CRC than older (≥ 55 years) individuals (HR: 0.404, 95%CI: 0.171–0.953) (Jurado-Fajardo et al. 2011).

CRC survival can be influenced by numerous factors that affect disease outcomes at the individual level. These factors range from body weight (Jayasekara et al. 2018) and race/ethnicity (Acuna-Villaorduna et al. 2021) to even socioeconomic status (Zhang et al. 2017). Lifestyle choices such as diet, physical activity, and smoking habits also play a crucial role (Jayasekara et al. 2018). Access to quality healthcare services are significant determinants as well (Muller et al. 2021). Additionally, psychological factors, including stress and mental disorders, can impact survival rates too (Lloyd et al. 2019). Therefore, understanding the interplay of these diverse elements is essential for interpreting studies’ findings and developing evidence-based comprehensive and effective treatment plans tailored to individual patient needs, regardless of age.

This study has some limitations. First, the data source for the survival analyses could represent a bias as it is possible that health insurance companies did not update the required information weekly as mandated by the government. And second, the database used for the vital status review includes records only for persons affiliated through two types of health insurance companies (public/subsidized and private/contributive), excluding individuals with other types of affiliations. Nevertheless, some strengths can be noted. To the best of our knowledge, this is the first study conducted in the city of Medellín, Colombia that compared survival outcomes between EOCRC and LOCRC and some related prognostic factors. Additionally, the large sample used in this study adds to the statistical significance of our findings.

## Conclusion

Among CRC patients from Colombia, EOCRC is associated with significantly better 5-year OS rates and prognosis compared to patients with LOCRC. Moreover, advanced stage and higher tumour grading are independent predictors of poorer OS. These findings highlight the importance of age-related risk stratification and personalized therapeutic approaches in CRC.

## Data Availability

No datasets were generated or analysed during the current study.
